# Respiratory virus immune response in the aged host

**DOI:** 10.1186/s12979-026-00559-7

**Published:** 2026-02-10

**Authors:** Olivia B. Parks, Anusha Kalavacharla, John V. Williams

**Affiliations:** 1 Department of Pediatrics, University of Pittsburgh School of Medicine, Pittsburgh, PA USA; 2 Program in Microbiology and Immunity, University of Pittsburgh School of Medicine, Pittsburgh, PA USA; 3 Department of Pediatrics, University of Wisconsin-Madison School of Medicine and Public Health, 1675 Highland Ave, Madison, WI 53792 USA

**Keywords:** Aged host, Immunity, Respiratory viral infections

## Abstract

Viruses are a major cause of acute respiratory illness in older adults and pose a substantial burden as the elderly population continues to grow. In the current COVID-19 global health crisis, achieving a better understanding of the aging immune system proves to be an imperative step in preventing and treating respiratory viral infections in older patients. Furthermore, many common respiratory viruses infecting older adults, including human metapneumovirus and parainfluenza virus, do not have licensed vaccines, thereby increasing the risk of severe infection in the aged host. Moreover, given the slowed immune response of older adults, vaccine efficacy for respiratory viruses such as influenza in older adults is minimal, indicating the need to develop more potent vaccines. A better understanding of the aging immune system would allow vaccines to target immunological deficits in the aged host. Three aspects of the aging immune system affect the response to respiratory viruses and vaccines: [1] innate immunity [2], the “inflammaging” hypothesis, and [3] the adaptive immune response. Several innate immune cells (neutrophils, macrophages, dendritic cells, and natural killer cells) as well as adaptive immune cells (T and B lymphocytes) exhibit significant functional impairment in older adults. The inflammaging hypothesis bridges the innate and adaptive arms of the immune system. This review aims to consolidate current knowledge and fill gaps in our understanding of the aged immune response to respiratory viruses.

## Impact of respiratory viral infections in the elderly

Respiratory viral infections (RVIs) in older populations are a major cause of morbidity and mortality, comprising 13–31% of all respiratory illnesses documented in this population [1–4]. By 2050, it is projected that adults > 60 years old will comprise 22% of the population worldwide [5], which will place a significant burden on global healthcare systems to care for this vulnerable population.

The top three families of virus that are the leading cause of lower respiratory tract infections in this population include: *Orthomyxoviridae*,* Picornaviridae*, and *Paramyxoviridae* [2, 3, 6],. Specifically, lower respiratory tract infections caused by influenza virus and RSV are leading causes of older adult patient hospitalizations, accounting for approximately 53,800 deaths annually in those 65 and older in the US [2, 3, 6]. Further, up to 90% of all influenza-related deaths in the US each year occur in the elderly [7]. The average direct medical costs in the US per season calculated from several influenza seasons came to approximately $10.4 billion, with 64% of this cost allocated to care for individuals 65 years and older [8]. RSV is linked to serious morbidity and mortality in care home residents, accounting for 12% of adult RSV hospital admissions. It was also found that the mortality rate in this group was 38%, significantly higher than the 3% seen in patients admitted from the community [9]. A systematic review and meta-analysis concluded that the in-hospital case fatality rate of respiratory syncytial virus (RSV) is 7.13% in adults older than 65 [10].

Rhinovirus and human metapneumovirus (HMPV) are also common causes of severe RVI affecting the aged population [11]. There is a significant burden of RVI in nursing homes, affecting 1.4–2.8/1000 resident days [1, 12]. One retrospective study of over 2000 patients hospitalized for RVI identified RSV in > 10% of individuals 65 and 8% among those 50–64 years [13, 14]. Another study of hospitalizations due to HMPV during nine winter seasons found that 46% of patients were individuals > 65yrs, while 35% were children < 2 years [15]. This study also concluded that HMPV and parainfluenza viruses (PIV) had similar prevalence, accounting for RVI that were not caused by RSV or influenza virus [15].

The COVID-19 pandemic was another example of how RVIs predominantly affect the elderly. At the beginning of the COVID-19 pandemic, patients 50 years and older comprised greater than 94% of COVID-19 related deaths [16]. A study reported that in 2020 alone, $6.3 billion were spent on Medicare costs for COVID-19-related hospitalizations [17]. A review of COVID-19 cases globally found that severe disease, hospitalization rates, and deaths were heavily skewed towards individuals over 65 [18]. In the US alone, nursing home residents only comprise 0.5% of the population, and yet represented 40% of COVID-19 deaths in the first year of the pandemic [18]. A recent study also found that HMPV and COVID-19 share similar clinical presentations, underscoring the importance of accurate diagnosis and testing for this vulnerable population [19]. Due to the atypical presentation and delay of RVI diagnosis in the elderly, antiviral therapy may also not be provided quickly enough [20]. A recent study found that the testing technique and whether multiple tests were used often improved the accuracy of detecting RSV in older adults [21]. For example, adding sputum PCR increased RSV infections detected in 52% of older adults while adding mouth/throat swab PCR testing increased RSV detected by 29% [21]. Overall, the type of testing and if multiple testing is used can impact whether RSV is detected in older adults and could indicate that RSV may be underreported in older adults due to inadequate testing. In addition, secondary bacterial infection is a common complication of RVI. One recent study found that 1/3 of older adults in the population study diagnosed with PCR confirmed RSV, also had bacterial pneumonia, making bacterial coinfection a significant cause of morbidity and mortality [22].

The current understanding of the aging immune system has identified changes in both innate and adaptive immune cells that lead to decreased function as observed by both in vitro and in vivo models. Leukocytes are rapidly dividing cells and therefore suffer from the genetic effects of aging, including genomic instability, epigenetic reconstruction, and telomeric deterioration [7]. Furthermore, immunosenescence and immune cell exhaustion are believed to lead to impaired leukocyte function. The culmination of this disequilibrium in inflammation and immune cell response leads to delayed viral clearance, viral dissemination to the lower respiratory tract, and an overall increased risk severe infection.

Taking these findings together, RVIs caused by viruses including influenza, COVID-19, RSV, HMPV, rhinovirus, and parainfluenza account for a significant portion of morbidity and mortality in older adults and warrant research to elucidate vaccination strategies and therapeutics to better care for this vulnerable population. To begin to address this gap, this review aims to understand three key factors that make up the aged immune response: (1) inflammaging and immunosenescence, (2) deficits in innate immunity, and (3) impaired adaptive immunity with the review ending on vaccine interventions utilizing trained immunity to better protect the aging population from RVIs. We will cover how functional changes to innate immune cells (neutrophils, macrophages, dendritic cells, and natural killer cells) as well as adaptive immune cells (T and B lymphocytes) contribute to an impaired immune response in older populations. The inflammaging hypothesis aims to link these impairments with the broader, systemic changes to the immune landscape of the aged host. Given the breadth of literature regarding aging, immunity, and RVIs, this review will focus on key cellular/molecular mechanisms in combination with the inflammaging hypothesis to better understand the aged host response to RVIs.

### Inflammaging & immunosenescence

**Fig. 1:**
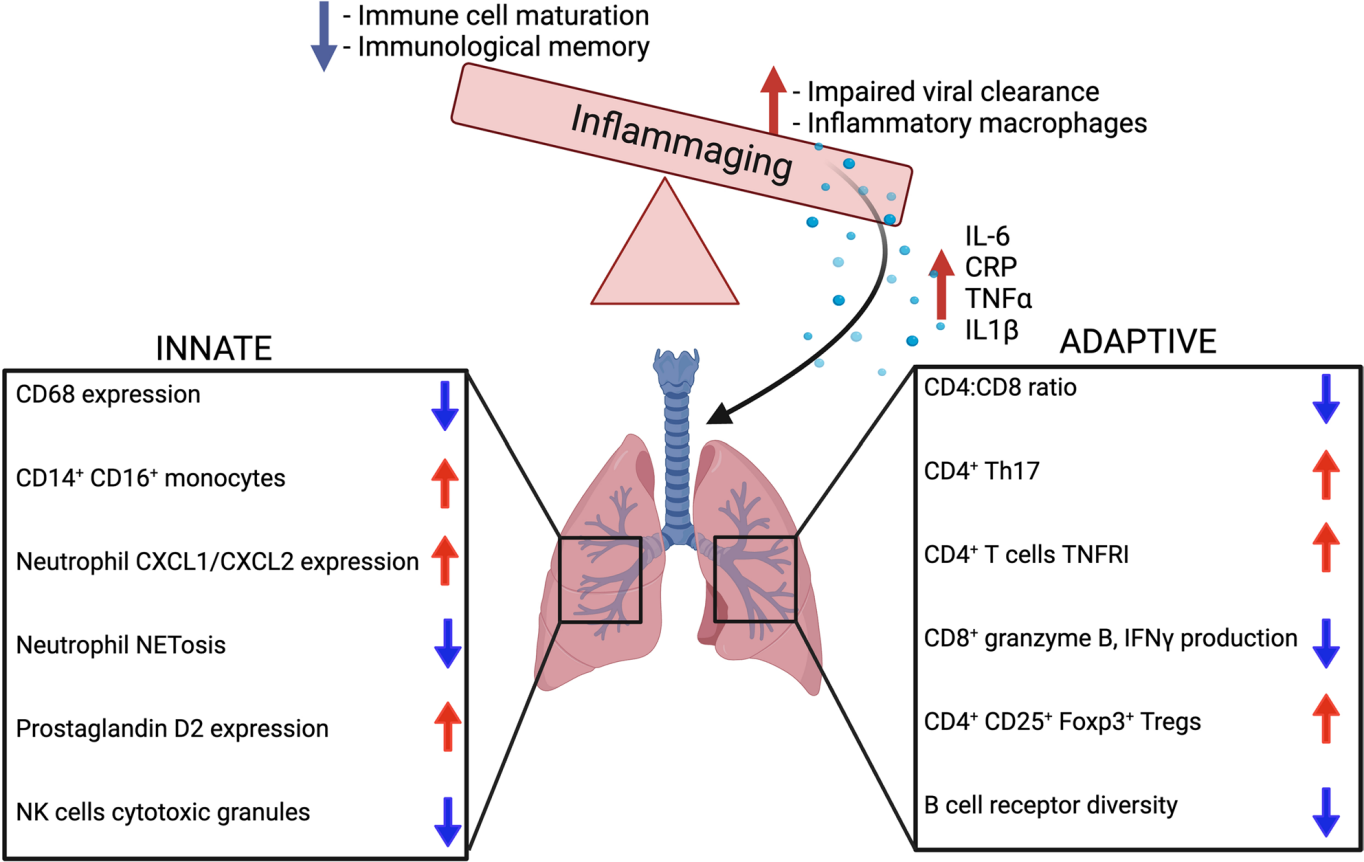
Summary of immune changes with increasing age. With increasing age there is a shift towards a pro-inflammatory state which has a detrimental effect on both the innate and adaptive immune systems. There is an overall decrease in innate immune cell function. Specifically, CD68 expression on monocytes and macrophages is downregulated with a subsequent increase in pro-inflammatory CD14+ CD16+ monocytes and CXCL1/CXL2 expression from neutrophils. Neutrophils also have impaired NETosis which prevents effective antimicrobial killing. Small molecule prostaglandin D2 expression is increased which contributes to innate immune cell dysfunction. NK cells also show impaired cytotoxic granule production. Concurrently, adaptive immune cells also exhibit significant impairment with a decreased CD4:CD8 ratio and upregulation of inflammatory CD4+ Th17 cells, CD4 TNFR1 expression, and CD4+ CD25+Foxp3+ Tregs. CD8+ T cell antiviral function is impaired with decreased production of cytotoxic cytokines such as granzyme B and IFNγ production. B cell receptor diversity is also diminished with increasing age which contributes to poor vaccine response in this age group

The “Inflammaging hypothesis” is a hypothesis used to describe cellular senescence [23] and refers to the phenomenon that with increased age there is a shift towards a pro-inflammatory state with upregulation of basal levels of pro-inflammatory cytokines (i.e. C-reactive protein (CRP), TNF, IL-1b, and IL-6) accompanied by an impaired anti-inflammatory response [23–25] (Fig. 1). It is posited that inflammaging is the “first hit” leading to a heightened proinflammatory state, which predisposes the aged individual to a second hit characterized by an absence of ‘robust’ and a presence of ‘frail’ immune response genes. This refers to the thought that there are some gene variants that are more susceptible to immunosenescence from chronic exposure to proinflammatory cytokines. This heightens the aged host’s susceptibility to infection or systemic disease [25]. This two-hit hypothesis helps explain the pathogenesis of multisystem conditions such as atherosclerosis, diabetes, Alzheimer’s, and most importantly for this discussion, immune dysfunction and increased risk of viral respiratory illnesses in the older adult [25]. In addition to this two-hit hypothesis comes a decrease in cortisol production and impaired function of the Hypothalamus-Pituitary-Adrenal (HPA) axis that shifts the balance from an anti-inflammatory to a pro-inflammatory response [26]. Chronic inflammation in the aged host is strongly connected with a greater incidence of chronic disease and decreased immune response to infection leading to increased mortality and prolonged hospital stay [27–30]. Studies in aged humans have shown that chronic inflammation is counteracted by anti-inflammaging cytokines such as IL-1Ra, IL-4, IL-10, and TGF-b1 [23]. When this balance between pro and anti-inflammatory cytokines is lost, inflammaging dominates and leads to an impaired immune response and increased susceptibility to RVIs.

Aged mice (> 18 months) have elevated levels of pro-inflammatory cytokines at baseline compared to young mice (2–6 months) [25, 27, 31]. This imbalance leads to chronic inflammation and multisystem effects [32]. Franceschi et al. suggests there is a threshold that varies between individuals for which the pro-inflammatory state is beneficial, promoting immune cell maturation and immunological memory [25, 33]. When the basal pro-inflammatory response surpasses this threshold, the beneficial effects of inflammation are lost, and the detrimental pathological effects of inflammation dominate. This promotes a shift towards more pro-inflammatory macrophages and further perpetuates an impaired antiviral response leading to severe RVI in the aged host.

Chronic viral infection of CMV can lead to an accumulation of cellular debris and chronic inflammation [33, 34]. CMV infection promotes a pro-inflammatory microenvironment as part of its pathogenesis, thus accelerating inflammaging and impaired cellular function [33, 35]. In the presence of the inflammaging microenvironment, innate immune receptors such as TLRs and NOD-like receptors (NLRs) have impaired function, leading to pathogens and damage unrecognized by the immune system [33, 36]. The prevalence of CMV infection increases with age and CMV infection is prolonged in the elderly [37]. CMV infection also reduces the number of naïve T cells and the CD4/CD8 ratio [37]. This immune receptor hyporesponsiveness likely contributes to the replication of viral respiratory pathogens in the aged host.

As previously described, with age, the inflammatory profile of monocytes and macrophages undergoes specific alterations including increased inflammatory gene expression and decreased functionality. A similar trend is seen with the immune cell first responders, neutrophils. The result of this diminished function not only impairs the aged host’s response to an RVI but also leaves them susceptible to further lung damage and bacterial co-infections. DCs in the older population exhibit decreased costimulatory molecule expression and cytokine production relative to their younger counterparts; likewise, aged NK cells have decreased cytokine and cytotoxic granule secretion. Taken together, these findings reveal a clear link between the aging host and immune impairment, increasing their vulnerability to infections and systemic disease. Studies are ongoing to determine whether the inciting factor is immune cell dysfunction and exhaustion that promote a pro-inflammatory environment or whether the pro-inflammatory environment facilitates immune cell dysfunction/exhaustion. Understanding this relationship will help to identify pathways that can be modulated or targeted to improve the immune response in the aged host.

### Aged deficits in innate and adaptive immunity

#### Innate immunity

Age-related changes during RVIs have been primarily documented in monocytes, macrophages, dendritic cells (DCs), and natural killer (NK) cells. Dysfunction of these innate immune cells contributes to delayed viral clearance, enhanced inflammation, and increased morbidity and mortality in the aged host. Research has shown a skew towards myeloid cell production in the bone marrow of aged mice [38]. RNAseq analysis on bone marrow stromal cells in aged mice revealed increased expression of pro-inflammatory genes such as IL-6 and IL-1b [38]. Pattern recognition receptors (PRRs) are located on both innate immune cells [39] and nonimmune cells, most notably epithelial and endothelial cells [27, 40, 41]. These receptors also exhibit diminished pathogen recognition upon aging. As an example, toll-like receptor (TLR) expression is decreased on aged antigen presenting cells (APCs) compared with their younger counterparts in both human and mice [27, 41–49]. Due to decreased expression of TLRs, the subsequent signaling cascades in immune cells and pro-inflammatory cytokines released are also reduced in the aged host [27, 44, 47, 50–52]. The sections below describe current research in aging innate immune cells during RVIs including monocytes and macrophages, neutrophils, DCs, innate lymphoid cells (ILCs), and natural killer (NK) cells. DCs and tissue resident macrophages are the first cell types to encounter the virus while circulating monocytes rapidly respond to cytokine signals sent from DCs and activated T lymphocytes to migrate to the lung at the site of infection. Given the early role these cell types play in initiating the innate immune response and activating T lymphocytes to drive the adaptive immune response, it is critical to understand their function in older individuals to determine if deficits in these cells contribute to severe respiratory disease seen in older adults.

#### Monocytes & macrophages

Expression of the monocyte lineage marker CD68 is decreased in bone marrow from aged mice, indicating a decrease in macrophage precursors [53, 54]. In concordance with this, scRNA-seq analysis in aged mice show reduced tissue-resident macrophages particularly in the lung [38]. There was no difference in circulating bone-marrow derived myeloid cells between age groups, but aged mice did have increased expression of inflammatory genes such as *Ccl5* and *Gdf15* [38]. Interferon (IFN)g-stimulated macrophages from aged mice expressed 50% less major histocompatibility complex (MHC) class II compared to young cells [53, 55]. Studies on circulating monocytes in the peripheral blood of elderly patients revealed that aged individuals also have increased CD14^+^ CD16^+^ pro-inflammatory monocytes in line with studies in aged mice [42, 56, 57]. When aged monocytes from elderly humans were stimulated with TLR4, they exhibited impaired phagocytosis and increased intracellular tumor necrosis factor (TNF), indicating impaired monocyte function [56]. Thus, in both aged mice and humans, monocytes and macrophages exhibit impaired signaling, MHC-II expression, and phagocytosis.

Alveolar and interstitial macrophages represent the first line of defense in the lung against RVIs [58] and are the primary producers of pro-inflammatory cytokines such as TNF, IL-6, and IL-8 that serve an essential role in controlling respiratory viruses such as RSV and influenza [58, 59]. Alveolar macrophages (AMs) are a self-renewing population of tissue-resident macrophages that serve primarily an anti-inflammatory role to phagocytose foreign particles and secrete anti-inflammatory cytokines such as TGF-b and IL-10 to control the inflammatory immune response by neighboring cells [60]. Interstitial macrophages (IMs) are a unique subset of macrophages derived from circulating monocytes [60]; IMs secrete immunoregulatory cytokines such as IL-10, present antigen via MHC-II, and control Th2 mediated allergic inflammation [60]. Influenza infection of aged mice showed a reduced number of macrophages localized to the lung as well as down-regulation of cell cycling pathways in AMs [61]. A recent study used heterochronic adoptive transfer and parabiosis techniques to determine that the lung microenvironment in the aged host may prevent AMs from proliferating during influenza infection, thus contributing to the delay in viral clearance [62].

Macrophage function is also affected by circadian rhythms; the importance of circadian rhythms in immune regulation is a well-established concept. Recent research by Blacher et al. reports that the circadian rhythm of macrophages in older adults impacts macrophage function especially in the context of respiratory viral infections [63]. Blacher et al. found that macrophages from aged individuals have decreased expression of Kruppel-like factor 4 (KLF4), which contributes to macrophages’ circadian clock [63]. This study suggests that age-associated downregulation of KLF4 in aged macrophages may inhibit diurnal trafficking of these cells from the bone marrow to sites of infection [63]. Furthermore, gene expression analysis revealed only 53 genes undergoing rhythmic transcription in aged macrophages compared to 680 genes in macrophages isolated from young mice [63]. This study found that people 65 and older who were carriers of certain KLF4 variants were more prone to developing infections and had increased mortality compared to younger, non-carrier individuals [63]. Further, a study comprising 276 adults > 65 + found that older adults mounted a stronger antibody response to the influenza vaccine when administered in the morning [64].

Taken together, there are distinct changes in the inflammatory profile and gene expression signature of monocytes and macrophages in the aged host, particularly in the lung microenvironment. This contributes to the increased susceptibility of elderly patients to respiratory viral infections.

#### Dendritic cells

There are two main subtypes of dendritic cells (DCs): conventional DCs (cDCs) and plasmacytoid DCs (pDCs). cDCs originate from the myeloid lineage, while pDCs are of mixed ontogeny. However, both DC sub-types accumulate in the lung during RVIs [58]. pDCs are one of the main producers of type I IFN and often activate IFNg-producing CD8^+^ T cells via antigen presentation and upregulation of co-stimulatory receptors such as CD80. cDCs can polarize to type 1 cDCs (cDC1) which promote the activation of antiviral cytotoxic CD8^+^ T cells [58].

In human subjects, DCs isolated from aged donors exhibited increased secretion of pro-inflammatory cytokines (i.e. IL-6, TNF, and metalloproteinase A) [65]. Studies have found that increased age is associated with impaired DC function, namely the ability to phagocytose and migrate to lymph nodes. When human pDCs were stimulated with influenza virus [66] or HSV-2 [67], decreased IFNa was observed in aged pDCs. Consequently, TLR7 and TLR9 expression was decreased and oxidative stress was notably increased [68]. These deficits in aged DC function impair their ability to effectively prime T cells and help mediate viral clearance by CD8^+^ T cells [27]. Studies in aged mice revealed an increase in prostaglandin D2 expression in the lung [69]. With increased PGD_2_ in the lung, there was impaired migration of DCs, which impacted antiviral T cell response due to lack of APCs [69]. When PGD_2_ was blocked, there was increased DC migration and enhanced antiviral T cell response specifically to SARS-CoV-2 [69, 70]. These studies identify a unique role for PGD_2_ in the aged lung and a mechanism by which to enhance the antiviral response to RVIs by actively recruiting more DC to the site of infection.

Several in vitro [27, 71–73] and in vivo studies with murine models [27, 74–76] found that the expression of costimulatory molecules (CD40, CD80, CD86) [27, 74, 77] and the production of cytokines were decreased in DCs from aged mice compared to their young counterparts. There was also a decrease in DC-specific/intracellular adhesion molecule type-3-grabbing nonintegrin (DC-SIGN), which is an essential component of the T lymphocyte stimulating pathway [73]. Grolleau-Julius et al. reported that DC migration and CCR7 chemokine signaling were impaired in DCs from aged mice upon B16-ovalbumin antigen stimulation [68, 73]. Collectively, these data indicate that aged DCs exhibited impaired maturation upon antigen stimulation, leading to diminished T cell responses.

#### Neutrophils

Neutrophil influx into the lung during RVI has been studied in both humans and mice. Neutrophil influx in these models varies between viruses, with the highest concentration of neutrophils infiltration found in rhinovirus [78], influenza A [78], and RSV infections [78, 79]. Neutrophil migration to the lungs has both positive and negative effects. Neutrophils are the most abundant immune cell present in lung infiltrate within the first 24 h post-RVI [58]. They are crucial in phagocytosing pathogens and secreting inflammatory chemokines that recruit other immune cells to the lung during infection [58]. Neutrophils have several stages to development (i.e. promyelocyte, myelocyte, metamyelocyte, band neutrophil, and segmented neutrophil) and may exist in circulation in any one of these stages, thus creating a heterogenous neutrophil population [80]. With age, mice have increased secretion of CXCL1 and CXCL2, which are chemokines that recruit neutrophils to tissues, which promotes basal neutrophil accumulation [80]. During RVIs, the population of neutrophils recruited to the lung changes dramatically and consists predominantly of immature neutrophils. However, the neutrophils recruited during RVIs in aged individuals exhibit decreased migration, NET production, and phagocytosis, impairing the antiviral immune response [80].

The timing of neutrophil migration to the lungs following a viral infection is crucial for antiviral response and lung repair following viral clearance [81, 82]. However, neutrophils represent a double-edged sword when it comes to viral clearance vs. causing further post-viral lung damage. There is a systemic increase in basal inflammatory cytokines that cause neutrophils to accumulate in the airways in the absence of infection in aged humans [83]. During an RVI in an older host, neutrophils are recruited in a disorganized way to the site of infection [83]. Subsequently, at resolution of infection, neutrophils are among the cell types that persist in the lung, perpetuating further lung damage after the virus has been cleared [83]. This phenomenon has been shown in aged mice post influenza infection [84] as well as in mice infected with HMPV and RSV where neutrophils were responsible for releasing damaging oxidative enzymes [58]. However, when neutrophils are depleted during HMPV infection in mice, there was significantly delayed viral clearance [58]. In addition, diminished neutrophil function in the aged host promotes bacterial co-infection [85]. In aged mice infected with influenza, neutrophils had impaired antimicrobial response, which enhancing pneumococcal bacterial colonization and co-infection [85].

In humans, neutrophils in older humans have increased apoptosis, impaired migration to the lung, and reduced ability to kill pathogens via NETosis [82]. Neutrophils in both aged humans and mice also exhibit decreased ability to produce pro-inflammatory cytokines and enzymes such as myeloperoxidase, elastase, IL-6, and IL-8, with a compensatory increase of anti-inflammatory markers including IL-10 and an antagonist of the IL-1 receptor [27, 48, 86–89]. Furthermore, in both models, neutrophils exhibited an impaired ability to form neutrophil extracellular traps (NETs) that function to trap and restrain invading pathogens [27, 87, 90–94]. Impaired function of neutrophils in aged patients recovering from influenza infection could contribute to their increased susceptibility to bacterial co-infections and severe pneumonia. Neutrophils from young donors were more responsive to the cytokine granulocyte-macrophage colony-stimulating factor (GM-CSF), which led to increased chemotaxis, but this effect was not observed in neutrophils isolated from elderly individuals. This indicates age-dependent differences in GM-CSF signaling in neutrophils, as detailed in [95]. Further studies in humans report an upregulation in aged neutrophils of Src homology domain-containing protein tyrosine phosphatase-1 (SHP-1) and suppressors of cytokine signaling (SOCS) [95, 96]. These inhibitory molecules can cause decreased function of Phosphoinositide 3-kinase (PI3K) and other downstream effectors of the GM-CSF signaling pathway, which could explain why neutrophils in the aged host exhibit decreased responsiveness to GM-CSF [95]. In addition, neutrophils from aged adults are more prone to apoptosis indicating a risk in the aged host for neutropenia [82, 95, 96].

Notably, although RSV bronchiolitis primarily affects children under the age of 2, neutrophils are believed to be the main driver of bronchiolitis pathogenesis during RSV infection in this age group [97]. Neutrophils are abundant in the airways of RSV bronchiolitis autopsy specimens and are thought to promote mucus and pro-inflammatory cytokine production [97]. This was further supported when depletion of neutrophils in mouse models of RSV bronchiolitis caused a decrease in pro-inflammatory cytokines (i.e. TNF, MPO, and MMP9) and also reduced mucus production [97]. Co-culturing neutrophils with RSV-infected airway epithelial cells resulted in increased apoptosis of airway epithelial cells [97].

Taken together, as first responders, neutrophils are crucial early in respiratory viral infections. With increasing age, not only does neutrophil function decline during RVIs, but the timing of neutrophil migration and subsequent apoptosis at resolution of RVIs is impaired, perpetuating further post-viral lung damage, decreased ability to kill pathogens, and increased susceptibility to bacterial co-infections.

#### Innate lymphoid cells (ILCs) and natural killer cells

Group 1 ILCs (ILC1) are part of the first line of defense against viral infections and some bacteria. Aged mice exhibit decreased ILC1 numbers, specifically NK cells, compared to their young counterparts in spleen, lungs, and liver [38]. There is a higher proportion of more differentiated CD56^dim^ NK cells and a decrease in immature CD56^bright^ NK cells [38]. This shift to CD56^dim^ NK cells may be in part due to cytomegalovirus (CMV) and Epstein-Barr virus (EBV) with primary infections usually happening early in life and leading to life-long latent infections [98]. Chronic viral infections such as CMV and EBV can promote expansion of defective CD56^dim^ NK cells, which can impair the innate immune response against acute viral infections (i.e. influenza, HMPV, RSV) [98]. Aged NK cells from humans also exhibit decreased cytokine and cytotoxic granule secretion, most notably in IFNg and perforin production, respectively [38, 98]. In NK cell transfer experiments between aged and young mice, the aged microenvironment impaired NK cell maturation and baseline homeostasis [38]. This suggests that there are age-related extrinsic changes in the aged host that promote NK cell impairment and decreased function.

*Guo* et al. examined the cross talk between DC and NK cells in viral infections of aged (> 18 months) and young mice (< 3 months) [99]. Aged DC cells exhibited an impaired ability to activate NK cells from aged mice, leading to decreased activation and cytokine secretion from NK cells. Aged DCs co-cultured with young NK cells stimulated less effectively than young DCs, leading to decreased IFNg secretion. Aged mice had significantly less CD69 expression and NK cell granzyme B secretion [99]. Collectively, these findings suggest that NK cells exhibit impaired activation and cytotoxic function in both aged humans and mice.

### Adaptive immunity

#### T lymphocytes

CD4^+^ T cells exhibit both intrinsic and extrinsic alterations in function with increasing age [103]. Intrinsically, aged CD4^+^ T cells have impaired T cell receptor (TCR) signaling with reduced ZAP-70 activation, decreasing IL-2 production, which leads to a diminished systemic cell-mediated response [104]. In addition, aged CD4^+^ T cells upregulate tumor necrosis factor receptor 1 (TNFRI) and TNFR-associated death domain protein (TRADD), Fas, and Fas ligand, all contributing to increased susceptibility to apoptotic death. One study found that CD4^+^ T cells contribute to the inflammaging profile often seen in older adults, with aged CD4^+^ T cells tending to display a Th17 phenotype and produce inflammatory cytokines (e.g. IL-6, IL-17 A, IL-17 F, IL-21, and IL-23); this phenotype was reversible by treating T cells with metformin ex vivo [105].

To study extrinsic effects on CD4^+^ T cell deficits associated with age, one study [106] transferred bone marrow from either young or old mice into an irradiated young or old host and allowed the bone marrow to reconstitute. They tracked young versus old CD4^+^ T cells to determine the proliferative and functional capacity of these cells [106]. They found that naïve CD4^+^ T cells that were exposed to the aged host microenvironment exhibited increased function regardless on the age of the donor [106]. These findings indicate that the functional deficits observed in aged T cells are due to intrinsic age-related changes within the cell and not related to extrinsic effects of the age of the host [106].

An important subset of CD4 T cells are the tissue resident CD4^+^ T cells. Recent studies have shown that with increasing age, there is a depletion of naïve CD4^+^ T cells with an accumulation of effector (CCR7^−^ CD45RA^−^) and central memory (CCR7^+^ CD45RA^−^) CD4^+^ T cell subsets [107, 108]. Across a lifespan, there is repetitive antigen stimulation, causing CD4^+^ T cell telomeres to have shortened telomeres due to refractory telomerase induction [107]. CD4^+^ T cells begin to demonstrate age-dependent demethylation, TCR restriction, diminished IL-2 production, loss of CD8 expression, and decreased production of cytotoxic cytokines, perforin and granzyme B, which are a characteristic of immunosenescence [107].

The relationship between increasing age and Treg function is complex and not well understood, but it is largely supported that CD4^+^CD25^+^Foxp3^+^ Tregs increase with age, contributing to immunosenescence and a diminished T effector cell response [109–111]. This leads to an increased risk for autoimmune diseases, cancer, and severe infections in older adults [112–114]. Johnstone et al. enrolled 1072 residents in nursing homes and found fewer circulating naïve CD8^+^ CD45RA^+^CCR7^+^ T cells but more CD8^+^CD45RA^+^CCR7^−^ memory T cells and senescent CD8^+^CD28^−^ T cells compared to healthy adults < 65 [1]. Similar results have been reported in aged mice [115]. Decreased chemokine CCR7 expression may partly explain why T cell migration and localization to secondary lymphoid organs is impaired in the aged host [116]. In addition, there were increased Tregs in the adults over 65 compared to younger adults, which parallels results of other studies [117, 118]. The increased number of CD4^+^CD25^hi^ Tregs in the aged host may contribute to the increase incidence and severity of RVI in older adults [1, 110, 118]. Garg et al. measured Treg function in 18–20 month old C57BL/6 mice compared to 3–4 month old C57BL/6 mice and found that CD4^+^ cells isolated from aged mice contained hypomethylated DNA and significantly increased Foxp3 expression [111]. Aged Tregs more effectively removed cysteine from the extracellular environment, preventing T effector cell proliferation, and aged mice had higher IL-10 levels. This data suggests a distinct phenotype of increased Treg number and suppressive function in the aged host. In addition, Tregs may represent a target to modulate the aged immune response to RVI [112].

### CD8^+^ T lymphocytes

Studies of human T cell response to influenza vaccine found that CD8^+^ T lymphocytes in the older adults failed to produce adequate levels of CD8^+^ effector cytokines, granzyme B, and IFNg [7, 119, 120]. The inhibitory receptor programmed cell death protein 1 (PD-1), which is associated with lung T cell impairment during RVI when co-expressed with other inhibitory receptors (e.g. T cell immunoglobulin and mucin-domain containing-3 (TIM-3) and lymphocyte-activation gene 3 (LAG-3)) [121, 122], was upregulated at baseline on T cells of uninfected, otherwise healthy aged mice compared to their young counterparts [123].

Although the CD8^+^ T cell population is markedly increased in the aged host, sometimes comprising as much as 70–80% of the total CD8^+^ T cell population, these cells had impaired T cell receptor diversity and impaired CD8^+^ T cell response in a murine influenza model [68, 124]. In addition, with increasing age, there is a decline in naïve T cells (T_N_) and an increase in virtual memory T cells (T_VM_) [125]. T_VM_ are a subset of antigen inexperienced CD8^+^ T cells that express high levels of CD44 and the receptors for IL-15 and IL-17 [125]. T_VM_ are rapid responders to antigen in young mice, but with increased age these T_VM_ exhibit decreased function and a senescence profile [125]. One group found that aged T_VM_ failed to proliferate upon stimulation, had impaired accumulation of cyclin D1, and increased expression of Bcl-2 in both in vitro and in vivo studies [125]. Furthermore, adoptive transfers of aged CD8^+^ T cells into young mice failed to recover the T_VM_ rapid response to antigen that was observed in young mice [125]. In addition, recent paper found that thefe is a distinct population of CD8^+^ T cells that express CD39 and CD73. This population of CD8 T cells accumulates in older adults and has been shown to promote tumor growth through the CXCL16-CXCR6 axis [126].

CD8^+^ memory T cells are also greatly affected by increasing age. In older adults, there is a significant decrease in naïve CD8^+^ T cells repertoire the accompanies thymic involuation [127]. Concurrently, there is an expansion of the CD8^+^ memory T cell compartment. However, there is a loss in diversity of this memory T cell compartment because the older host is unable to generate many new resident memory cells due to the lack of a sufficient naïve CD8^+^ T cell population [127]. Specifically, in the lung, memory CD8^+^ T cells can show a robust accumulation, but lack adequate cytotoxic and antiviral function [127]. This contributes to the impaired antiviral response to the viral URIs and also impacts vaccine efficacy.

Together, these findings support that there are significant deficits in both CD4 and CD8 T lymphocyte populations as well as their subsets with increasing age. Vaccines and/or therapies targeting this immune cell compartment may prove to be effective at prevention and treatment of RVI in the aged host.

### Trained immunity & potential interventions

Trained immunity is a rapidly growing field of research that aims at understanding immunologic memory with an emphasis on identifying deficits in establishing immunological memory to vaccines [128]. Trained immunity goes hand in hand with innate immune cell interactions with B cells that help promote long term memory and illicit an antibody response to vaccines. Therefore, it is crucial to study B cell function in older adults and identify new vaccine strategies to target deficits in trained immunity with increasing age in order to improve vaccine efficacy in this vulnerable population.

Studies have found that in conjunction with inflammaging, there are substantial epigenetic and metabolic changes that occur in innate immune cells, particularly monocytes, macrophages, and NK cells [129]. Upon each encounter with pathogens, these innate immune cells are reprogrammed via long noncoding RNAs (lnRNAs) and modifications from histones (i.e. H3K4me3) to upregulate glycolysis and oxidative phosphorylation [129]. When the same pathogen is encountered again, these modified innate immune cells are able to respond more quickly and robustly to the pathogen. Studies have shown that certain vaccines can be used to confer a wider protection against multiple pathogens [128, 130]. One study found that treated mice with β-glucan protected mice against multiple bacterial pathogens known to cause pneumonia, enteritis, and peritonitis by induing monocyte proliferation and production of IL-1β [128, 131].

Aged humans exhibit decreased number of both naïve and circulating mature B cells, decreased diversity of B cell receptors (BCR) available for activation, impaired antigen specific production of antibodies, and altered production of memory B cells [7]. Chronic inflammation results in an accumulation of age-associated B cells (ABCs). ABCs express T-bet, CD11b, and CD11c and their development depends on pro-inflammatory cytokines, IL-21 and IFN$\:\gamma\:$ as well as TLR7 signaling [132, 133]. With the accumulation of ABCs comes increased risk of autoimmunity and depletion of follicular B cells, thus contributing to impaired germinal center (GC) formation [132–134].

Follicular dendritic cells have decreased function, which leads to impaired B cell activation and antibody production [135]. Moreover, the aged host has impaired GC formation with a decreased ratio of T follicular helper (T_FH_) to T follicular regulatory (T_FR_) [136]. This leads to diminished function of T_FH_, which prevents adequate GC formation [136]. This phenomenon could also contribute to why aged individuals exhibit a poor humoral response after vaccination [137, 138].

When Henry et al. administered influenza vaccine to young and older adults the group found that B cells from aged hosts had a fixed repertoire, indicating that B cells were not undergoing somatic hypermutation upon activation by the vaccine [139]. In contrast, young adult B cells showed increased diversity with continuous accumulation of hypermutations in the Fab region, resulting in higher specificity to influenza antigen [139]. Studies from Frasca and Blomberg also showed that upon administration of the influenza vaccine to older humans there was an impaired vaccine-specific antibody response [140–142]. One study proposed that relative levels of DNA methylation were partially responsible for the lack of vaccine responsiveness in older adults [140, 143].

Gibson et al. determined that somatic hypermutation of the Fab antibody region in the GC of lymph nodes occurs at a similar rate in young and older adults [144]. However, there was a substantial decrease in IgH-V complementarity during the initial recombination that occurs to generate the BCR, indicating a loss in diversity in the aged B cells [144]. Since B cells play a vital role in both humoral immunity and antigen presentation to T cells, the loss in BCR diversity leads to a subsequent loss in ability to recognize distinct antigens and contributes to the hyporesponsiveness of both B and T cells to RVI in the aged host. In addition, Frasca and Blomberg identify a population of pro-inflammatory double negative B cells that expand substantially with increased age [140, 145, 146]. This population of B cells have a characteristic phenotype of exhausted memory or tissue-like memory B cells (CD19^+^ CD27^−^ IgD^−^) [140, 147, 148]. These tissue-like memory B cells have been found in younger adults with autoimmune [140, 147–149] and infectious disease [140, 150, 151], which indicates that they expand during a pro-inflammatory state, aligning with the inflammaging in older adults.

Re-evaluating and re-designing vaccine strategies for older adults is imperative to improve morbidity and mortality from respiratory viral illnesses. For example, the current vaccine strategy for the influenza vaccine includes injecting subcutaneously or mucosal intradermally for optimal penetration [7]. The influenza vaccine itself consists of quadrivalent strains as opposed to the previously used trivalent strains in order to achieve a wider coverage [7]. The vaccine also contains increased number of influenza antigens coupled with protein adjuvants, such as MF59 and ASO3, which promote a more robust immune response [7, 152, 153]. HMPV vaccination strategies in aged mice showed that aged mice mounted a poor IgG antibody response in response to re-challenge 5 wks post UV-inactivated vaccination [154]. Other studies suggest targeting vaccine strategies at the level of B cell populations – naïve antigen specific B cells, memory B cells, and plasma cells – to fully optimize vaccine delivery and immune responsiveness [155]. Modulating doses of vaccine and improving vaccine bioavailability may help recruit and activate more naïve B cells [155]. Furthermore, enhancing plasma cell access to bone marrow microenvironments may increase plasma cell function, survival, and differentiation to improve antibody titers [155]. Perfecting the vaccine booster schedule may promote a more robust memory B cell response to ensure long-lasting immunity [155]. Mannick et al. shows in a phase 2 clinical trial that administration of an mTOR inhibitor to older adults caused an increase in antiviral gene expression with a subsequent improved antibody response to the influenza vaccine [156]. This group also found that mTOR inhibitor treatment also reduced PD-1 expression on CD4 and CD8 T lymphocytes, indicating a promising role for mTOR inhibitors in enhancing vaccine responsiveness in older adults [157].

Overall, trained immunity and vaccine strategies are key players in understanding RVI in the aged host. Current aging research is now focused on utilizing trained immunity to alleviate chronic inflammation and improve immune response to RVIs. These proposed vaccine strategies may enable physicians to circumvent vaccine unresponsiveness in the older patient, particularly in light of the recent COVID vaccines, ultimately leading to more complete coverage and protection against these devastating viral respiratory infections.

### T lymphocytes

Aging has a negative impact on several functions of T lymphocytes including impaired T cell response and Th1 polarization to viral respiratory antigens presented by APC, decreased circulating naïve T cells, increased anergic and senescent T cells, impaired T cell receptor diversity, and increased virtual memory CD8^+^ T cell populations [7].

One study used a GFP marker to tag recent thymic emigrants to measure the CD4:CD8 ratio in young and aged mice. They found that the CD4:CD8 ratio negatively correlated with the age of the mouse [68, 100]. By 70–80 years of age, a significant depletion of CD4^+^ effector T cells accompanied by a prolonged half-life of CD8^+^ memory T cells was seen, causing the latter to accumulate in the aged host [68, 101]. One possible explanation for this finding involves increased antiapoptotic Bcl-2 expression, which stabilizes the mitochondrial membrane in CD8^+^ T cells, preventing cytochrome c from leaking out of the mitochondria and inducing apoptosis [68]. There is also increased production of type I IFN and resultant downstream signaling, which could impact the half-life of CD8^+^ T cells [68]. A third explanation considers that chronic antigen stimulation over the course of an individual’s life could contribute to the accumulation of CD8^+^ memory T cells [68]. Thymic involution occurs with advanced age, which may contribute to the decreased number of new naïve CD45RA^+^ T cells in the aged host [68, 100, 102]. Taken together, this depletion of naïve CD4^+^ and CD8^+^ T lymphocytes in the aged host increases their susceptibility to pathogens and contributes to their impaired adaptive immune response.

#### CD4+ T lymphocytes

CD4+ T cells exhibit both intrinsic and extrinsic alterations in function with increasing age [103]. Intrinsically, aged CD4+ T cells have impaired T cell receptor (TCR) signaling with reduced ZAP-70 activation, decreasing IL-2 production, which leads to a diminished systemic cell-mediated response [104]. In addition, aged CD4+ T cells upregulate tumor necrosis factor receptor 1 (TNFRI) and TNFR-associated death domain protein (TRADD), Fas, and Fas ligand, all contributing to increased susceptibility to apoptotic death. One study found that CD4+ T cells contribute to the inflammaging profile often seen in older adults, with aged CD4+ T cells tending to display a Th17 phenotype and produce inflammatory cytokines (e.g. IL-6, IL-17A, IL-17F, IL-21, and IL-23); this phenotype was reversible by treating T cells with metformin ex vivo [105].

To study extrinsic effects on CD4+ T cell deficits associated with age, one study [106] transferred bone marrow from either young or old mice into an irradiated young or old host and allowed the bone marrow to reconstitute. They tracked young versus old CD4+ T cells to determine the proliferative and functional capacity of these cells [106]. They found that naïve CD4+ T cells that were exposed to the aged host microenvironment exhibited increased function regardless on the age of the donor [106]. These findings indicate that the functional deficits observed in aged T cells are due to intrinsic age-related changes within the cell and not related to extrinsic effects of the age of the host [106].

An important subset of CD4 T cells are the tissue resident CD4+ T cells. Recent studies have shown that with increasing age, there is a depletion of naïve CD4+ T cells with an accumulation of effector (CCR7- CD45RA-) and central memory (CCR7+ CD45RA-) CD4+ T cell subsets [107, 108]. Across a lifespan, there is repetitive antigen stimulation, causing CD4+ T cell telomeres to have shortened telomeres due to refractory telomerase induction [107]. CD4+ T cells begin to demonstrate age-dependent demethylation, TCR restriction, diminished IL-2 production, loss of CD8 expression, and decreased production of cytotoxic cytokines, perforin and granzyme B, which are a characteristic of immunosenescence [107].

The relationship between increasing age and Treg function is complex and not well understood, but it is largely supported that CD4+CD25+Foxp3+ Tregs increase with age, contributing to immunosenescence and a diminished T effector cell response (109–111). This leads to an increased risk for autoimmune diseases, cancer, and severe infections in older adults (112–114). Johnstone et al enrolled 1072 residents in nursing homes and found fewer circulating naïve CD8+ CD45RA+CCR7+ T cells but more CD8+CD45RA+CCR7- memory T cells and senescent CD8+CD28- T cells compared to healthy adults <65 [1]. Similar results have been reported in aged mice [115]. Decreased chemokine CCR7 expression may partly explain why T cell migration and localization to secondary lymphoid organs is impaired in the aged host [116]. In addition, there were increased Tregs in the adults over 65 compared to younger adults, which parallels results of other studies [117, 118]. The increased number of CD4+CD25hi Tregs in the aged host may contribute to the increase incidence and severity of RVI in older adults [1, 110, 118]. Garg et al measured Treg function in 18−20 month old C57BL/6 mice compared to 3–4 month old C57BL/6 mice and found that CD4+ cells isolated from aged mice contained hypomethylated DNA and significantly increased Foxp3 expression [111]. Aged Tregs more effectively removed cysteine from the extracellular environment, preventing T effector cell proliferation, and aged mice had higher IL-10 levels. This data suggests a distinct phenotype of increased Treg number and suppressive function in the aged host. In addition, Tregs may represent a target to modulate the aged immune response to RVI [112].

#### CD8+ T lymphocytes

Studies of human T cell response to influenza vaccine found that CD8+ T lymphocytes in the older adults failed to produce adequate levels of CD8+ effector cytokines, granzyme B, and IFNg [7, 119, 120]. The inhibitory receptor programmed cell death protein 1 (PD-1), which is associated with lung T cell impairment during RVI when co-expressed with other inhibitory receptors (e.g. T cell immunoglobulin and mucin-domain containing-3 (TIM-3) and lymphocyte-activation gene 3 (LAG-3)) [121, 122], was upregulated at baseline on T cells of uninfected, otherwise healthy aged mice compared to their young counterparts [123].

Although the CD8+ T cell population is markedly increased in the aged host, sometimes comprising as much as 70–80% of the total CD8+ T cell population, these cells had impaired T cell receptor diversity and impaired CD8+ T cell response in a murine influenza model. [68, 124]. In addition, with increasing age, there is a decline in naïve T cells (TN) and an increase in virtual memory T cells (TVM) [125]. TVM are a subset of antigen inexperienced CD8+ T cells that express high levels of CD44 and the receptors for IL-15 and IL-17 [125]. TVM are rapid responders to antigen in young mice, but with increased age these TVM exhibit decreased function and a senescence profile [125]. One group found that aged TVM failed to proliferate upon stimulation, had impaired accumulation of cyclin D1, and increased expression of Bcl-2 in both in vitro and in vivo studies [125]. Furthermore, adoptive transfers of aged CD8+ T cells into young mice failed to recover the TVM rapid response to antigen that was observed in young mice [125]. In addition, recent paper found that thefe is a distinct population of CD8+ T cells that express CD39 and CD73. This population of CD8 T cells accumulates in older adults and has been shown to promote tumor growth through the CXCL16-CXCR6 axis [126].

CD8+ memory T cells are also greatly affected by increasing age. In older adults, there is a significant decrease in naïve CD8+ T cells repertoire the accompanies thymic involuation [127]. Concurrently, there is an expansion of the CD8+ memory T cell compartment. However, there is a loss in diversity of this memory T cell compartment because the older host is unable to generate many new resident memory cells due to the lack of a sufficient naïve CD8+ T cell population [127]. Specifically, in the lung, memory CD8+ T cells can show a robust accumulation, but lack adequate cytotoxic and antiviral function [127]. This contributes to the impaired antiviral response to the viral URIs and also impacts vaccine efficacy.

Together, these findings support that there are significant deficits in both CD4 and CD8 T lymphocyte populations as well as their subsets with increasing age. Vaccines and/or therapies targeting this immune cell compartment may prove to be effective at prevention and treatment of RVI in the aged host.

### Trained immunity & potential interventions

Trained immunity is a rapidly growing field of research that aims at understanding immunologic memory with an emphasis on identifying deficits in establishing immunological memory to vaccines [128]. Trained immunity goes hand in hand with innate immune cell interactions with B cells that help promote long term memory and illicit an antibody response to vaccines. Therefore, it is crucial to study B cell function in older adults and identify new vaccine strategies to target deficits in trained immunity with increasing age in order to improve vaccine efficacy in this vulnerable population.

Studies have found that in conjunction with inflammaging, there are substantial epigenetic and metabolic changes that occur in innate immune cells, particularly monocytes, macrophages, and NK cells [129]. Upon each encounter with pathogens, these innate immune cells are reprogrammed via long noncoding RNAs (lnRNAs) and modifications from histones (i.e. H3K4me3) to upregulate glycolysis and oxidative phosphorylation [129]. When the same pathogen is encountered again, these modified innate immune cells are able to respond more quickly and robustly to the pathogen. Studies have shown that certain vaccines can be used to confer a wider protection against multiple pathogens [128, 130]. One study found that treated mice with β-glucan protected mice against multiple bacterial pathogens known to cause pneumonia, enteritis, and peritonitis by induing monocyte proliferation and production of IL-1β [128, 131].

Aged humans exhibit decreased number of both naïve and circulating mature B cells, decreased diversity of B cell receptors (BCR) available for activation, impaired antigen specific production of antibodies, and altered production of memory B cells [7]. Chronic inflammation results in an accumulation of age-associated B cells (ABCs). ABCs express T-bet, CD11b, and CD11c and their development depends on pro-inflammatory cytokines, IL-21 and IFNγ as well as TLR7 signaling [132, 133]. With the accumulation of ABCs comes increased risk of autoimmunity and depletion of follicular B cells, thus contributing to impaired germinal center (GC) formation (132–134).

Follicular dendritic cells have decreased function, which leads to impaired B cell activation and antibody production [135]. Moreover, the aged host has impaired GC formation with a decreased ratio of T follicular helper (TFH) to T follicular regulatory (TFR) [136]. This leads to diminished function of TFH, which prevents adequate GC formation [136]. This phenomenon could also contribute to why aged individuals exhibit a poor humoral response after vaccination [137, 138].

When Henry et al administered influenza vaccine to young and older adults the group found that B cells from aged hosts had a fixed repertoire, indicating that B cells were not undergoing somatic hypermutation upon activation by the vaccine [139]. In contrast, young adult B cells showed increased diversity with continuous accumulation of hypermutations in the Fab region, resulting in higher specificity to influenza antigen[139]. Studies from Frasca and Blomberg also showed that upon administration of the influenza vaccine to older humans there was an impaired vaccine-specific antibody response (140–142). One study proposed that relative levels of DNA methylation were partially responsible for the lack of vaccine responsiveness in older adults [140, 143].

Gibson et al determined that somatic hypermutation of the Fab antibody region in the GC of lymph nodes occurs at a similar rate in young and older adults [144]. However, there was a substantial decrease in IgH-V complementarity during the initial recombination that occurs to generate the BCR, indicating a loss in diversity in the aged B cells [144]. Since B cells play a vital role in both humoral immunity and antigen presentation to T cells, the loss in BCR diversity leads to a subsequent loss in ability to recognize distinct antigens and contributes to the hyporesponsiveness of both B and T cells to RVI in the aged host. In addition, Frasca and Blomberg identify a population of pro-inflammatory double negative B cells that expand substantially with increased age [140, 145, 146]. This population of B cells have a characteristic phenotype of exhausted memory or tissue-like memory B cells (CD19+ CD27- IgD-) [140, 147, 148]. These tissue-like memory B cells have been found in younger adults with autoimmune (140, 147–149) and infectious disease [140, 150, 151], which indicates that they expand during a pro-inflammatory state, aligning with the inflammaging in older adults.

Re-evaluating and re-designing vaccine strategies for older adults is imperative to improve morbidity and mortality from respiratory viral illnesses. For example, the current vaccine strategy for the influenza vaccine includes injecting subcutaneously or mucosal intradermally for optimal penetration [7]. The influenza vaccine itself consists of quadrivalent strains as opposed to the previously used trivalent strains in order to achieve a wider coverage [7]. The vaccine also contains increased number of influenza antigens coupled with protein adjuvants, such as MF59 and ASO3, which promote a more robust immune response [7, 152, 153]. HMPV vaccination strategies in aged mice showed that aged mice mounted a poor IgG antibody response in response to re-challenge 5 wks post UV-inactivated vaccination [154]. Other studies suggest targeting vaccine strategies at the level of B cell populations – naïve antigen specific B cells, memory B cells, and plasma cells – to fully optimize vaccine delivery and immune responsiveness [155]. Modulating doses of vaccine and improving vaccine bioavailability may help recruit and activate more naïve B cells [155]. Furthermore, enhancing plasma cell access to bone marrow microenvironments may increase plasma cell function, survival, and differentiation to improve antibody titers [155]. Perfecting the vaccine booster schedule may promote a more robust memory B cell response to ensure long-lasting immunity [155]. Mannick et al shows in a phase 2 clinical trial that administration of an mTOR inhibitor to older adults caused an increase in antiviral gene expression with a subsequent improved antibody response to the influenza vaccine [156]. This group also found that mTOR inhibitor treatment also reduced PD-1 expression on CD4 and CD8 T lymphocytes, indicating a promising role for mTOR inhibitors in enhancing vaccine responsiveness in older adults [157].

Overall, trained immunity and vaccine strategies are key players in understanding RVI in the aged host. Current aging research is now focused on utilizing trained immunity to alleviate chronic inflammation and improve immune response to RVIs. These proposed vaccine strategies may enable physicians to circumvent vaccine unresponsiveness in the older patient, particularly in light of the recent COVID vaccines, ultimately leading to more complete coverage and protection against these devastating viral respiratory infections.

## Discussion

RVIs take a significant toll on the older adult population, especially during the COVID-19 pandemic. Therefore, it is imperative to understand the interaction of the aging immune system with viruses to better care for patients. With aging comes a multitude of changes in both the innate and adaptive arms leading to an impaired response to RVI. There are several potential mechanisms that contribute to these immunological changes, including impaired innate cell function, inflammaging, the chronic production of pro-inflammatory mediators, T lymphocyte anergy, and diminished B cell repertoire. The multifactorial nature of aging makes research in this field complex and poses significant challenges, which makes it difficult to identify just one cell type or pathway to target that will improve the aged immune response to RVIs.

Senolytics as mechanisms to improve the immune response in older adults and target the multifactorial nature of the aging immune system is an emerging field. One study found treating mice infected with SARS-CoV-2 with dasatinib and quercetin decreased mortality and ameliorated clinical symptoms throughout infection [158]. Other studies have investigated using senolytics to clear senescent cells in aged mice as a mechanism to reverse the effects of age-related diseases including atherosclerosis [159, 160] and type 2 diabetes [159, 161]. Senolytics have been used to remove senescence-induced changes in CD4 T cells in aged mice infected with influenza [159, 162]. Specifically, aged mice infected with influenza were treated with a senolytic that blocked TGF-b, a known product of senescent cells. By blocking TGF-b, the CD4 T cell repertoire was restored to that of young mice with a significant decrease in Foxp3^+^ CD4^+^ T regulatory cells [162]. Overall, there is growing evidence that senolytics are a valuable agent to target and eliminate age-related immune dysfunction.

As described above, the fields of immunology and gerontology have made significant steps towards understanding the aging immune system in the context of RVIs. However, the aged host’s response to viral respiratory infections is still incompletely understood as is the ability to effectively diagnose and treat patients with these infections. Furthermore, the poor memory response to vaccination and resistance to therapies, such as PD-1 blockade, underscores the growing need to discover ways to modulate the immune system in the aged host in order to enhance the immune response to viral respiratory pathogens. Additional information on the immune response during an RVI could enable the creation of more effective vaccines and establish preventative measures to lessen the detrimental impact these infections have on older adults. Importantly, the strides made towards establishing vaccines against common RVIs (e.g., RSV, HMPV) for adults over 65 years old could lead to an effective vaccine for other high-risk populations including young children and the immunocompromised. Further research in the field will provide a deeper understanding of the complex mechanisms surrounding the aging immune system, RVI pathogenesis, and provide a better quality of life for older patients.

## Data Availability

No datasets were generated or analysed during the current study.

## Abbreviations

RVI: Respiratory viral infections
RSV: Respiratory syncytial virus
HMPV: Human metapneumovirus
PIV: Parainfluenza viruses
DC: Dendritic cells
NK: Natural killer cells
PRRs: Pathogen recognition receptors
TLR: Toll-like receptor
APC: Antigen presenting cells
TNF: Tumor necrosis factor
IFN: Interferon
MHC: Major histocompatibility complex
LPS: Lipopolysaccharide
IL: Interleukin
Jmjd3: Jumonji domain-containing protein D3
AM: Alveolar macrophages
GM-CSF: Granulocyte-macrophage colony-stimulating factor
SHP-1: Src homology domain-containing protein tyrosine phosphatase-1
SOCS: Suppressors of cytokine signaling
PI3K: Phosphoinositide 3-kinase
NET: Neutrophil extracellular traps
pDC: Plasmacytoid dendritic cells
cDC: Conventional dendritic cells
DC-SIGN: Dendritic cell-specific intercellular adhesion molecule-3-grabbing non-integrin
CRP: C-reactive protein
CMV: Cytomegalovirus
NLR: NOD-like receptor
TCR: T cell receptor
TNFRI: Tumor necrosis factor receptor 1
TRADD: TNFR-associated death domain protein
PD-1: Programmed cell death protein 1
TIM-3: T cell immunoglobulin and mucin-domain containing-3
LAG-3: Lymphocyte-activation gene 3
T_N_: Naïve T cells
T_VM_: Virtual memory T cells
BCR: B cell receptor
ABC: Age-associated B cell
GC: Germinal center
T_FH_: T follicular helper cell
T_FR_: T follicular regulatory cell
